# Exploring the Current Landscape of Intravenous Infusion Practices and Errors (ECLIPSE): protocol for a mixed-methods observational study

**DOI:** 10.1136/bmjopen-2015-009777

**Published:** 2016-03-03

**Authors:** Ann Blandford, Dominic Furniss, Imogen Lyons, Gill Chumbley, Ioanna Iacovides, Li Wei, Anna Cox, Astrid Mayer, Kumiko Schnock, David Westfall Bates, Patricia C Dykes, Helen Bell, Bryony Dean Franklin

**Affiliations:** 1 UCL Interaction Centre, London, UK; 2 Centre for Medication Safety and Service Quality, Imperial College Healthcare NHS Trust, London, UK; 3 Research Department of Practice and Policy, UCL School of Pharmacy, London, UK; 4 UCL Medical School, University College London, London, UK; 5 Brigham and Women's Hospital, Boston, Massachusetts, USA

**Keywords:** AUDIT

## Abstract

**Introduction:**

Intravenous medication is essential for many hospital inpatients. However, providing intravenous therapy is complex and errors are common. ‘Smart pumps’ incorporating dose error reduction software have been widely advocated to reduce error. However, little is known about their effect on patient safety, how they are used or their likely impact. This study will explore the landscape of intravenous medication infusion practices and errors in English hospitals and how smart pumps may relate to the prevalence of medication administration errors.

**Methods and analysis:**

This is a mixed-methods study involving an observational quantitative point prevalence study to determine the frequency and types of errors that occur in the infusion of intravenous medication, and qualitative interviews with hospital staff to better understand infusion practices and the contexts in which errors occur. The study will involve 5 clinical areas (critical care, general medicine, general surgery, paediatrics and oncology), across 14 purposively sampled acute hospitals and 2 paediatric hospitals to cover a range of intravenous infusion practices. Data collectors will compare each infusion running at the time of data collection against the patient's medication orders to identify any discrepancies. The potential clinical importance of errors will be assessed. Quantitative data will be analysed descriptively; interviews will be analysed using thematic analysis.

**Ethics and dissemination:**

Ethical approval has been obtained from an NHS Research Ethics Committee (14/SC/0290); local approvals will be sought from each participating organisation. Findings will be published in peer-reviewed journals and presented at conferences for academic and health professional audiences. Results will also be fed back to participating organisations to inform local policy, training and procurement. Aggregated findings will inform the debate on costs and benefits of the NHS investing in smart pump technology, and what other changes may need to be made to ensure effectiveness of such an investment.

**Strengths and limitations of this study**

- Intravenous medication errors are common and efforts are required to reduce their occurrence. Little is known about practices and how they relate to the prevalence of error in England.
- Smart infusion pumps are beginning to be adopted, but their potential impact on the prevalence of error needs to be assessed.
- A mixed-methods (quantitative and qualitative) observational study will investigate different intravenous practices and how they relate to prevalence of errors.
- The study is limited to medication administration errors. It does not include prescription errors or evaluate the clinical appropriateness of what is prescribed.

## Background

### Safety of intravenous medication administration

Intravenous medication is essential for many hospital inpatients. However, providing intravenous therapy is complex, and errors in the administration of intravenous medication are common. In a systematic review of UK studies using structured observation of medication administration, errors were five times more likely in intravenous than non-intravenous doses.^1^ Published error rates vary from 18% to 173% of intravenous doses given.^2^ An international systematic review estimated the probability of making at least one error in the preparation and administration of a dose of intravenous medication to be 0.73, with most errors occurring at the reconstitution and administration steps.^3^ While many of these errors do not result in patient harm, all can cause anxiety for staff and patients, and reduce patients’ confidence in their care. As a result, the administration of intravenous medication has been identified as a significant topic of concern by regulators, manufacturers and healthcare providers.^4^

### The potential role of smart infusion pumps

To reduce errors associated with intravenous infusions, ‘smart pumps’ incorporating dose error reduction software have been widely advocated.^5–7^ This software checks programmed infusion rates against preset limits for each drug and clinical location, using customisable ‘drug libraries’, to reduce the risk of infusion rates that are too high or too low. Limits may be ‘soft’ (in which case they can be over-ridden following confirmation by the clinician) or ‘hard’ (in which case they cannot). Smart pumps may be stand-alone or integrated with electronic prescribing and/or barcode administration systems, and usually allow administrative data such as number and types of over-rides to be downloaded for analysis. While smart pumps were in use in 68% of US hospitals in 2011,^8^ their use is not yet as widespread in the UK.^9^ Such technology can potentially identify and prevent some kinds of medication errors but cannot prevent all possible errors. Smart pump use also comes at a cost, both financial and in terms of changes to practice needed to make their use effective. For instance, Husch *et al*^10^ carried out a hospital-wide point prevalence study of errors in intravenous infusions using standard infusion pumps and identified infusion rate errors in 37 cases (8% of all infusions), and wrong medication in 14 cases (3%). However, they estimated that only one of these errors would have been prevented using stand-alone smart pumps. More were judged to be potentially preventable if the pumps were integrated with other hospital systems, such as electronic prescribing and barcode-assisted administration.

A recent systematic review identified 21 quantitative studies of smart pumps,^11^ the majority of which studied the over-rides recorded in the smart pump logs and/or used unreliable methods of identifying medication errors and adverse drug events such as incident reports. The authors concluded that smart pumps can reduce but not eliminate error, and that the picture was far from conclusive. Furthermore, most studies were conducted in the USA, none was from the UK where systems for prescribing and administering medication differ from those in the USA.^12^ For example, nurses play a more active role in preparing intravenous medication in the UK, and all medication orders have to be in writing. We therefore know little about the effect on patient safety of using smart pumps in general and nothing about their likely impact in the UK.

### Methodological approach

To supplement a quantitative description of the prevalence, types and clinical importance of errors, qualitative methods help to understand why errors occur and why technologies designed to deliver safer practices do not always do so. Interviews and observational methods can also shed light on the complex and varied situations within which infusion devices are used across clinical contexts and hospitals.^13^ ^14^ Studies have found that infusion practices vary significantly between and within hospitals. For example, in a series of observational studies, nurses in an oncology day care unit were found to follow fairly basic procedures in setting up planned infusions,^15^ whereas nurses in an intensive care unit routinely used advanced functionality, frequently setting up several pumps in parallel to deliver different medications.^16^ There is an increasing drive towards standardising devices within institutions, intended to reduce potential risks associated with staff using a range of different devices or devices configured in different ways, or being required to operate devices that they are not familiar with or have not been trained to use. However, not all clinical areas require the same functionality—for example, Carayon *et al*^17^ studied how nurses use infusion devices in different areas of the hospital. They compared the tasks actually carried out with the tasks as defined by ward protocol. They identified divergences in practice and highlighted ways in which these divergences increased overall system vulnerability.

Few previous studies have brought together the perspectives of quantitative observational studies with qualitative interviews and observations. Furthermore, this multidisciplinary project combines clinical and human factors research perspectives. Our approach is to deliver overview and detail of intravenous medication infusion practices and identify the roles of different infusion technologies and practices in minimising the risks of medication administration errors.

## Methods and analysis

### Aim and objectives

The Exploring the Current Landscape of Intravenous Infusion Practices and Errors (ECLIPSE) study is a multiphase project, the aims of which are to describe how intravenous medication is infused in English hospitals, how often and why errors occur and the likely impact of smart pump technology on patient safety (table 1).

1. This protocol relates to the first phase, which will comprise a point prevalence study of intravenous infusion administration and qualitative focus groups and interviews with key hospital staff. Subsequent phases will comprise in-depth observational studies from a human factors perspective, to situate the findings of the first phase within a broader context, and a synthesis of the findings to make recommendations for safer administration of intravenous medication. Objectives of the first phase are to document the prevalence, types and clinical importance of errors involving the infusion of intravenous medication in a sample of English hospitals, and compare findings with data from a US study.
2. Explore potential sources of variation in the rates, types and clinical importance of errors in relation to mode of infusion delivery (gravity administration, standard infusion pumps and syringe drivers, and smart infusion pumps and syringe drivers) and clinical area (critical care, general surgery, general medicine, paediatrics and oncology).
3. Explore staff perspectives on potential explanatory factors behind the findings.
4. Describe, based on interviews with hospital staff, how intravenous infusions are administered in a sample of English hospitals, focusing on differences in practices, equipment, policies and processes, within and between hospitals.

**Table 1:** The three different phases of the ECLIPSE study

1	Point prevalence study and follow-up focus groups and interviews in 16 hospital sites	▸ Quantitative observational study of intravenous administration ▸ In-depth discussions with key staff at participating sites to understand practices
2	In-depth observational study in a subset of these sites	More detailed ethnographic study of a subset of sites to better understand detailed practices
3	Recommendations and summary reporting	Dialogue with participating hospitals to jointly identify and communicate recommendations for best practice

ECLIPSE, Exploring the Current Landscape of Intravenous Infusion Practices and Errors.

### Study design

The study will employ a mixed-methods approach bringing together complementary quantitative and qualitative methods to address these objectives. A quantitative point prevalence study, using observational methods, will be used to document the prevalence, types and clinical importance of errors associated with the infusion of intravenous medication. Qualitative focus groups and interviews with relevant hospital staff will help explain the findings.^18^ The design of the point prevalence study is based closely on that used in a similar US multicentre study which studied general medical, general surgical, medical intensive care and surgical intensive care units (K Schnock and DW Bates, personal communication, 2013). This approach, originally developed by Husch *et al*,^10^ involves trained staff systematically comparing details of each intravenous infusion in progress at the time of observation with the medication prescribed, to identify any discrepancies. Within ECLIPSE, once quantitative data have been analysed, interviews with key staff will also be conducted to reflect on the point prevalence results and details of hospital intravenous practices.

### Study setting, recruitment and sample selection

The study will take place within acute hospitals across England. Expressions of interest were sought from English National Health Service (NHS) hospital trusts through the National Institute for Health Research (NIHR) Clinical Research Network, the UK National Association of Medical Device Educators and Trainers (NAMDET) and contacts from a previous study.^9^ Interested parties were then invited to complete an online survey to provide an overview of their hospital, capacity to take part, infusion pumps and practices. Eighteen NHS trusts responded to the survey, providing information about 26 potential hospital sites. We are inviting 14 acute hospitals and 2 specialist children's hospitals to take part in the first phase of ECLIPSE. Hospitals are being chosen purposively, with the aim of representing maximum variation in terms of type, size, geographic location, potential indicators of patient safety such as being a Bruce Keogh Trust,^19^ hospital mortality indexes^20^ and media reports, and their self-reported use of infusion devices and smart pump technology. We aim to include a mixture of hospitals that use smart pumps with dose error reduction software, and those who do not. If any particular gaps are identified, additional hospital sites will be approached to ensure a wide range of practices are included.

In each participating hospital, we will study a minimum of three clinical areas (critical care, general medicine and general surgery). This set has been chosen to represent a range of clinical areas while also mirroring as closely as possible the US study, recognising that UK hospitals do not typically have separate medical and surgical intensive care units. However, in contrast to the US study, we will also include paediatric and oncology, since these are areas where, at least anecdotally, different types of errors may be more likely to occur and/or have greater consequences. In each hospital, each clinical area may include just one, or more than one, individual ward as needed.

We aim to include observation of 2100 infusions in total across all study sites. Using nQuery Advisor (Statistical Solutions, V.7.0), this sample will give a CI around a 10% overall error rate across hospitals and clinical areas of 8.7–11.3%.^7^ ^10^

### Definitions

Medication infusions will be taken to include any medication, fluids, blood products and nutrition administered via intravenous infusion, including patient-controlled analgesia.

A medication administration error will be considered to be any deviation in the administration of an intravenous infusion from a doctor's written medication order, the hospital's intravenous policy and guidelines, or the manufacturer's instructions. This will be taken to include the administration of medication to which the patient had a documented allergy or sensitivity; other aspects of the clinical appropriateness of the medication order and its administration will not be assessed. We will also collect data on other procedural or documentation discrepancies which do not meet the definition of a medication administration error but which may increase the likelihood of administration errors occurring. These will include patients not wearing an identification wristband with the correct information, tubing not being tagged and labelled in accordance with local policy, and failure to document the administration of the medication in line with hospital policy. Definitions of error and discrepancy types are given in table 2.

**Table 2:** Definitions of error and discrepancy types

Discrepancy/error type	Definition
Medication administration errors	
Unauthorised medication/fluids (no documented order)	Fluids/medications are being administered but no medication order is present. This includes failure to document a verbal order if these are permitted as per hospital policy.
Wrong medication or fluid	A different fluid/medication/diluent as documented on the intravenous bag (or bottle/syringe/other container) is being infused compared with that specified on the medication order or in local guidance.
Concentration discrepancy	An amount of a medication in a unit of solution that is different from that prescribed.
Dose discrepancy	The same medication but the total dose is different from that prescribed.
Rate discrepancy	A different rate is being delivered from that prescribed. Also refers to weight-based rates calculated incorrectly including using a different patient weight from that recorded on the patient's chart.
Delay of dose or medication/fluid change	An order to change the medication or rate not carried out within 4 h of the written medication order, or as per local policy.
Omitted medication or intravenous fluids	The medication prescribed was not administered.
Allergy oversight	Medication is prescribed/administered despite the patient having a documented allergy or sensitivity to the drug concerned.
Expired drug	The expiry date/time on either the manufacturer's or additive label has been exceeded.
Roller clamp discrepancy	The roller clamp is not positioned appropriately/correctly.
Procedural and documentation discrepancies	
Patient identification error	Patient either has no identification (ID) band on wrist, or information on their ID band is incorrect.
Wrong or missing information on additive label	Any incorrect or missing information on the additive label, as required by hospital policy
Tubing not tagged according to policy	Tagging or labelling of tubing is different (either missing or incorrect) from requirements in hospital policy.
Documentation error	Medication/fluids administered but not documented correctly on chart, eg, missing signature, start time, etc.

### Data collection

#### Point prevalence study

At each study site, these data will be collected by two clinicians who are employees of the participating site, usually a nurse and a pharmacist or experienced pharmacy technician, following training by the research team. The training will include the study protocol, data collection and documentation procedures.

Data will be collected on 1 day or an equivalent period in each clinical area. On selected observation days, the local data collectors will move systematically around each ward, gathering data from each patient with an infusion running. The aim will be to gather data from every infusion that is being administered at that time. Wards will be selected with the aim of gathering data from 30 to 40 infusions from each clinical area in each hospital. The data collectors will compare the medication being administered against the patient's prescribed medication and relevant medication administration records to identify any discrepancies. This will include a comparison of the medication or fluid name, the concentration and rate of infusion. Relevant data such as patient allergies, pump type and any procedural or documentation errors will also be recorded. Where it appears that one discrepancy has been introduced to compensate for another discrepancy, data gatherers are being trained to note this. Data collectors are also expected to have familiarised themselves with their hospital's policies and to check guidelines and manufacturers’ instructions for themselves when in doubt. The two observers will work together, each checking the data collected with the other and agreeing whether or not any medication administration error or other discrepancy has been identified. Multiple errors and discrepancies may be identified for a single infusion.

#### Contextual interviews

Once data from the point prevalence study have been analysed, we will liaise with key staff in participating hospitals to share our findings and explore potential explanatory factors behind those findings (eg, nursing practices, equipment, policies and processes, staff management, training and competency assessment). This will involve a two-way dialogue with relevant members of staff including ward managers, senior nursing staff, patient safety specialists, medical electronics personnel, trainers, those with responsibility for procurement, and senior managers. We will invite a purposive sample of these staff (typically 3–5 per hospital) to participate in individual or group semistructured qualitative interviews, aiming for maximum variation in staff roles and responsibilities. The interview will focus on the participant's views of local policy and practice, what works well and less well, and what changes are under consideration. They will be used to clarify any queries from the observational data and will focus primarily on normal practice rather than extreme events. If there appear to be inconsistencies between the ways that data gathering has been done across sites (despite consistent training), this will also be explored during interviews and data will be adjusted if deemed necessary to improve consistency across sites. Subject to participants giving consent, interviews will be recorded and professionally transcribed.

### Data management and analysis

#### Point prevalence study

**Figure 1:**
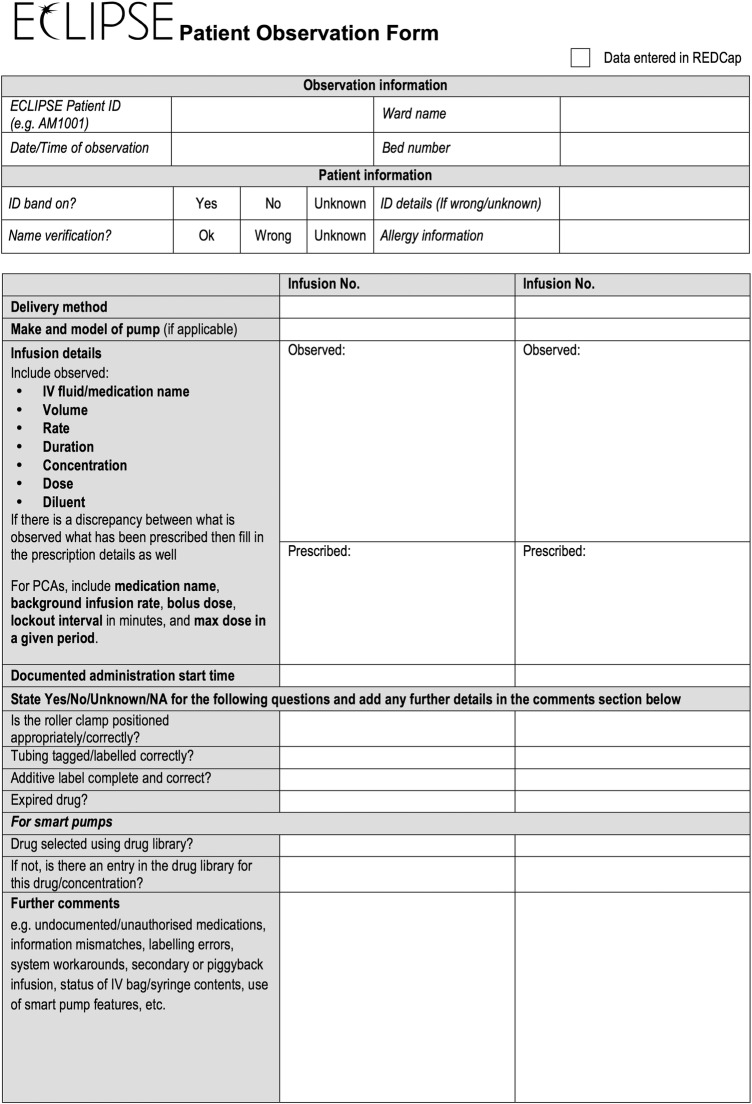
ECLIPSE patient observation form. ECLIPSE, Exploring the Current Landscape of Intravenous Infusion Practices and Errors.

Data will be either recorded on a standardised paper form (figure 1) and subsequently uploaded to a secure web-based REDCap data collection tool^21^ or entered directly into REDCap, depending on which better fits local practices. No patient-identifiable data will be entered. The data collection forms and online database have been adapted from that used in the USA and piloted to ensure they are applicable and relevant in the UK hospital context. Each site will be able to access their own data, while the central research team will be able to access data across all sites.

We will use two methods to assess the clinical importance of each discrepancy or error identified. First, these will be classified according to an adapted version of the US National Coordinating Council for Medication Error Reporting and Prevention (NCC MERP) index for categorising medication errors. The adaptation allows for the assigned severity ratings to be based on the likelihood of the error to have resulted in patient harm if it had not been intercepted, rather than actual patient harm for which the NCC MERP index was originally designed (tables 3 and 4). Our adapted version of NCC MERP also includes the term ‘discrepancy’ to capture minor deviations in practice where observers feel the deviation is not necessarily wrong and does not fall into our definition of a medication administration error. The second is separating category A into A1 and A2 so that more minor discrepancies can be better characterised. Guidance will be provided to data collectors on how to categorise deviations and errors, including illustrative examples. Where there are factors that make initial categorisation difficult, final assignment of clinical importance will be determined by consensus among the research team and local data collectors.

**Table 3:** NCC MERP index for categorising medication errors^23^

Harm	Category	Description
No error	A	Circumstances or events that have the capacity to cause error
Error, no harm	B	An error occurred but did not reach the patient
	C	An error occurred that reached the patient but did not cause patient harm
	D	An error occurred that reached the patient and required monitoring to confirm that it resulted in no harm to the patient and/or required intervention to preclude patient harm
Error, harm	E	An error occurred that may have contributed to or resulted in temporary harm to the patient and required intervention
	F	An error occurred that may have contributed to or resulted in temporary harm to the patient and required initial or prolonged hospitalisation
	G	An error occurred that may have contributed to permanent harm to the patient
	H	An error occurred that required intervention necessary to sustain patient life
Error, death	I	An error occurred that may have contributed to or resulted in the patient's death

NCC MERP, National Coordinating Council for Medication Error Reporting and Prevention.

**Table 4:** Adapted NCC MERP index that will be used in the ECLIPSE study

Harm	Category	Description
No error	A1	Discrepancy but no error
	A2	Capacity to cause error
Error, no harm	B	An error occurred but is unlikely to reach the patient
	C	An error occurred but is unlikely to cause harm despite reaching the patient
	D	An error occurred that would be likely to have required increased monitoring and/or intervention to preclude harm
Error, harm	E	An error occurred that would be likely to have caused temporary harm
	F	An error occurred that would be likely to have caused temporary harm and prolonged hospitalisation
	G	An error occurred that would be likely to have contributed to or resulted in permanent harm
	H	An error occurred that would be likely to have required intervention to sustain life
Error, death	I	An error occurred that would be likely to have contributed to or resulted in the patient's death

ECLIPSE, Exploring the Current Landscape of Intravenous Infusion Practices and Errors; NCC MERP, National Coordinating Council for Medication Error Reporting and Prevention.

Second, we will use an established method for assessing the severity of medication administration errors, developed and validated in the UK,^22^ which involves four experienced healthcare professionals, each assessing each error on a scale of 0–10, where 0 represents an error with no potential consequences to the patient and 10 an error which would result in death. The mean score across the four judges is then used as an index of severity which has been shown to be reliable and valid. Use of the two different methods for assessing clinical importance will allow us to compare the classifications obtained using NCC MERP with the scores obtained using the more time-consuming but potentially more robust second method. Using the adapted NCC MERP index will also permit comparison with the US study findings.

Descriptive statistics will be used to calculate overall medication administration error and discrepancy rates in relation to hospital site, clinical area and type of pump. The rate will be calculated in two ways: as number of discrepancies and errors divided by number of infusions observed and the proportion of infusions that involved at least one discrepancy or error; 95% CI will also be provided. Following the approach established by Husch *et al*,^10^ we will not perform more complex multivariate analyses but will compare different clinical contexts and different factors in intravenous medication administration—for example, critical care versus other wards, and use of smart pumps versus traditional ones. The aim will be to use the quantitative analysis as a basis for better understanding contributing causal factors, through subsequent focus groups, interviews and in-depth observations. An international comparison with the US data will also be carried out.

#### Contextual interviews

We will follow an inductive approach using thematic analysis.^24^ ^25^ Codes will be identified in the transcripts, broader themes will be recognised from these codes and codes may be related to each other through hierarchies and network diagrams. NVivo (QSR International, V.10) data analysis software will be used to facilitate analysis by multiple researchers. Interviews and analysis will be iterative so that subsequent interviews can take advantage of what is learnt from earlier interviews. Relevant theory will be used to inform additional deductive analysis if it provides leverage to interpret the data and explore the findings more deeply.^26^

## Ethics and dissemination

### Ethical considerations

This study has been approved by an NHS Research Ethics Committee (14/SC/0290) and site-specific R&D approval will be sought from each participating trust.

#### Data protection, informed consent and addressing observed errors

At a local level, the point prevalence study is comparable to an audit of intravenous medication practice for each site. Written informed consent will be sought from ward managers by the local co-ordinator before including ward areas in the study. Patients and ward staff will be offered an information sheet outlining the study. Feedback from a patient and public involvement workshop has been incorporated into the patient information sheet and the training of data collectors in how to approach and inform patients and their visitors about the study. As per the protocol approved by the ethics committee, written consent will not be required from individual patients or ward staff, and ward staff will not be notified of the timing of data gathering, to minimise the likelihood of this influencing their behaviour. Results from individual sites will be kept secure and anonymous to other sites.

Written informed consent will be sought from staff taking part in the qualitative interviews. Interviews will be audio-recorded and stored on secure password-protected computers before leaving the site. Interviews will be transcribed by a professional transcribing service, with due attention to confidentiality, and the transcripts anonymised.

If an error with potential to cause harm is identified during the point prevalence study, the local data collectors will inform the relevant nursing staff caring for the patient in a discreet manner so that remedial action can be taken in line with local procedures.

### Dissemination

This study will generate insights into current practice and the prevalence, causes and clinical importance of medication administration errors and other procedural and documentation discrepancies concerning intravenous infusions, which we anticipate will be of interest to and have implications for a variety of stakeholders. Dissemination will take place throughout the project.

The most immediate beneficiaries of our research will be the patients and staff of participating hospitals. To achieve rapid local impact, we will discuss findings with key staff in each hospital and deliver a tailored written report relating to each individual site, which participating trusts will be able to use to inform local policy, training and procurement.

We will also liaise with relevant national and international groups, such as NHS England, NAMDET, the Medusa IV Medicines Guide^27^ and the Infusion Systems Safety Initiative, hosted by the Association for the Advancement of Medical Instrumentation, to aid further dissemination and translation of the implications of our findings into practice. In later phases of the project, we will also hold workshops with healthcare professionals, manufacturers and other stakeholders to share our findings and refine our recommendations. We aim to influence the international academic community, as well as practising clinicians, through conference presentations and peer-reviewed publications.

Finally, besides disseminating our findings through published documents and professional domains, we will engage with the broader public through social media channels such as blogs, Twitter and YouTube. We will develop patient-facing summaries of our results and recommendations and take advice from patient representatives through planned patient and public involvement workshops on other means of sharing findings with the public.

## Results: pilot and initial site

Two of the authors have trialled the data collection form and REDCap Data Entry at their hospital. This provided an opportunity for practical learning. For example, to speed data entry, we streamlined the data entry tool and now advise observers to enter data in parallel, side by side, so they are able to discuss error classification together in an efficient manner. To help ensure consistency across sites, a document giving examples of errors and discrepancies, and suggested ratings of likely harm, will be updated and circulated to data collectors as the study progresses. Anonymised pilot data will be shown to participating sites as an example of what to expect in terms of data collection and data entry.

Learning from the initial participating site has also led to minor modifications to training materials and data collection procedures. For example, we have created a ‘Hints, Tips and Frequently Asked Questions’ sheet for local data collectors to share knowledge between sites, which will be updated and circulated as the study progresses. Data collectors at the first site shared informative short qualitative accounts of many discrepancies with the study team via email to give the observed errors and discrepancies appropriate context. This has now been formally incorporated into the data collection tool.

## Study status

Point prevalence data collection is ongoing. Data analysis and interviews will follow.

## Discussion

We have described a mixed-methods study comprising observation and interview in which we aim to determine the frequency and types of errors that occur in the infusion of intravenous medication, and to explore the range of infusion practices and the contexts in which errors occur. The point prevalence methodology described has a number of inherent limitations. First, the study focuses on medication administration, and the clinical appropriateness of the intravenous medication prescribed is not assessed. Data collection will capture infusions at one point in time and it is unlikely that errors in the preparation of a drug will be picked up. The hospitals taking part in this study are not randomly selected, and it is possible that they may differ in some way from hospitals that have not been approached or have chosen not to take part. In addition, as different observers at each participating hospital collect data, there may be variability between sites. Observers could make data entry errors. Arguably, transcription errors are less likely where they are entered directly into REDCap as it reduces the data entry steps; however, data can be checked against paper forms where they are used. To reduce the risk of errors in transcribing, we advise data collectors to enter the data soon after gathering it so that the observations are still fresh in their minds. We also advise data collectors to enter the data together to reduce the likelihood of error. Entering the data subsequently also allows additional time for reflection and discussion regarding the likely severity of errors, which may not be possible at the patient's bedside.

Adapting a protocol used in a US study will facilitate an international comparison. However, we are aware that cultural differences will need to be considered when comparing data from different countries.^12^ ^28^ Our study also has some differences in focus in that we are sampling different intravenous infusion practices such as gravity feed, non-smart pumps and smart pumps, rather than focusing solely on smart pumps, and a wide range of hospitals. We are also adding a focus on developing an explanation for the prevalence of error related to different intravenous practices from a human factors perspective.

We have experienced difficulties recruiting English hospitals that use smart pump technologies outside critical care. This is not surprising given Iacovides *et al*'s^9^ UK study in which less than 40% of their respondents use dose error reduction software in any clinical areas, and only a small proportion of these use it outside of critical care. At least three of our recruited sites have expressed an interest in doing a pre-smart and post-smart pump implementation study, using the same data collection methods, since they anticipate introducing smart pump technology across their site in the near future. This could lead to extensions to this protocol and modification to the phases described in table 1. It also indicates that this study's results will be timely as more English hospitals are considering whether to introduce smart pump technology. Sites not considering implementing smart pump technology have also shown keen interest in learning more about the performance of their intravenous infusion practices, highlighting the importance of this area. We anticipate that the findings will result in recommendations for future best practice; depending on the nature of those recommendations, this may lead to a future study to assess the impact of recommended interventions.
